# The Development of a Non-Pneumatic Tire Concept Based on a Fiber-Reinforced Epoxy Composite

**DOI:** 10.3390/polym17040505

**Published:** 2025-02-15

**Authors:** Jonathan Andrä, Tales de Vargas Lisboa, Axel Spickenheuer

**Affiliations:** 1 Leibniz-Insitut für Polymerfoschung Dresden e.V., Hohe Straße 6, 01069 Dresden, Germany; spickenheuer@ipfdd.de; 2 Hochschule für Technik und Wirtschaft Dresden, Fakultät Design, Friedrich-List-Platz 1, 01069 Dresden, Germany

**Keywords:** non-pneumatic tire (NPT), airless tire design, deformable spoke structures, lightweight tire design, fiber-reinforced polymer (FRP)

## Abstract

This paper investigates the use of glass and carbon fiber-reinforced polymer composites with epoxy matrices for non-pneumatic tires (NPTs), as an alternative to conventional elastomer-based designs. A novel NPT design approach was developed in three steps: (i) a finite element model with isotropic material properties was constructed to identify suitable spoke geometries; (ii) an anisotropic parametric study quantified key parameters influencing the load-bearing capability of two selected concepts from step (i); and (iii) a preferred version was chosen from step (ii) and evaluated under multiple load cases to ensure it met all requirements. The final tire design incorporates thick spiral spokes superimposed with a cosine-like function, showcasing the strengths and limitations of non-elastomeric reinforced polymers for NPT design. This study provides innovative insights into reducing the mass of NPTs and demonstrates the potential of fiber-reinforced polymer composites to achieve more lightweight, durable, and efficient NPT designs in comparison to pneumatic ones.

## 1. Introduction

**Figure 1:**
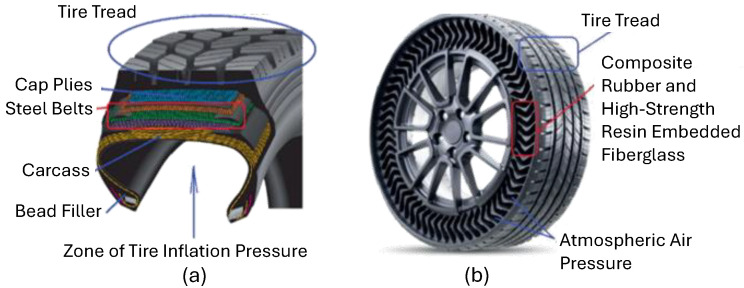
Structural comparison of radial pneumatic tire (**a**) and Unique Puncture-proof Tire System (UPTIS) (**b**) (modified from ref. [17]).

The significance of tires in automobiles is paramount, as they are the sole components responsible for transmitting forces between the vehicle and the ground [1,2,3]. Consequently, tires have a profound influence on various characteristics of cars, prompting intensive research in this domain [4,5,6,7]. This research focus is particularly pronounced for flexible non-pneumatic tires (NPTs) [8,9,10,11]. As an example, a structural comparison of a radial pneumatic tire and an NPT is given in Figure 1. Noteworthy advantages attributed to flexible NPTs, as compared to traditional pneumatic tires, include reduced rolling resistance [12], an extended lifespan [13], puncture resistance, the alleviation of design constraints on wheel assemblies, and a general decoupling of tire properties [14]. Collectively, these advantages provide a compelling economic and ecological rationale for the ongoing research and development of NPTs.

**Figure 2:**
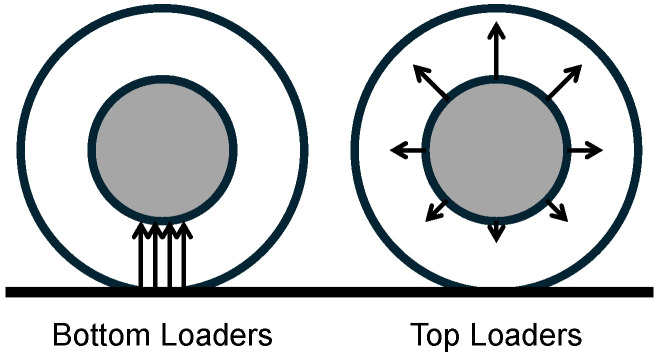
Load-carrying mechanics of tires based on [14].

To comprehend the evolution of NPTs, it is imperative to consider the historical context. Rhyne and Cron [14] investigated the advantages of pneumatic tires over the stiff non-pneumatic wheels that were prevalent before John Dunlop’s invention of the pneumatic tire in 1888 [15]. Their findings underscore multiple advantages of pneumatic tires. One of those is their low contact pressure, approximately equivalent to their inflation pressure. This characteristic facilitates high coefficients of friction, prolonged wear, and diminished surface damage to roads. In contrast, stiff NPTs exhibit exceedingly high contact pressure. Rhyne and Cron proposed a potential solution to mitigate this issue: the incorporation of a “shear beam”. They argued that rigid isotropic outer rings mimic the behavior of a bending beam, resulting in line pressures (at the initiation and conclusion of the contact patch). The shear beam concept involves two rigid inextensible membranes as the outer and inner rings, with a material possessing a low shear modulus between them. Gasmi et al. [16] researched this subject further, developing a comprehensive two-dimensional model of a top-loading NPT. Top-loading behavior, also observed in pneumatic tires, is generally preferred for NPTs over bottom-loading configurations; see Figure 2. Bottom loading means the load or force is supported from beneath the wheel hub. Top loading means the load or force is transferred through the outer ring of the tire to the top and applied to the hub from there.

Gasmi et al.’s [16] research revealed that a higher ratio of bending stiffness to shearing stiffness in the outer ring yields more uniform contact pressure for top loaders. However, certain NPTs, such as the Resilient Technologies NPT featuring honeycomb spokes, diverge from the conventional shear beam design [18]. Aboul-Yazid et al. [18] modeled both cases, with and without a shear layer. They found that the introduction of a shear layer does not decrease contact pressures across all spoke concepts, likely due to their predominant nature as bottom loaders. Consequently, recognizing the potential complexities introduced by shear beams in NPT designs, this paper intentionally excludes their incorporation to streamline the design process.

A common challenge in contemporary NPT designs is the considerable stress endured by most concepts over repeated use. To address this issue, the main objective of this paper is to evaluate the use of continuous fiber reinforcement, aiming to significantly improve the tire strength while simultaneously reducing the overall weight. A novel approach was considered within this work and is described below.

i. Different NPT spoke shapes were proposed, and both qualitative and quantitative evaluations, considering a material with isotropic behavior, were conducted to select well-performing concepts;
ii. Deeper analyses were performed of the selected concepts, in which the constructive parameters (number of spokes and thickness) and anisotropic behavior from the carbon and/or glass fiber orientation were taken into account;
iii. A new set of load conditions was then applied to the final concept, and the performance was evaluated.

Such an approach addressed the different responses of the NPT spoke shapes and how they are affected by their geometry, thickness, material behavior, etc. Therefore, this broad analysis of NPTs is to be conceived of as an initial step towards a novel non-elastomeric NPT, utilizing fiber-reinforced polymers (FRPs)—carbon/glass fiber-reinforced polymers (CFRPs/GFRPs)—in contrast to an elastomeric material approach. A carbon fiber-reinforced epoxy wheel has already been manufactured by Sandberg [13,19].

It will be shown and discussed in the next sections that the geometry of the spokes, thicknesses of the outer ring and spokes, material, and orientation play an important role in the mechanical response of the NPT as well as in its weight. Furthermore, this paper introduces a preliminary study that investigated the sensitivities of the parameters that were evaluated for a more complex and dynamic approach.

## 2. Materials and Methods

The dimensions of the tire under investigation, as well as other crucial specifications, such as the vertical stiffness and peak loads, were based on a Formula Student (Elbflorace Formula Student Team from the Technical University of Dresden) car forces evaluation based on ref. [20]. The results are detailed in Table 1. The *Hoosier FSAE 16.0X7.5-10* pneumatic tire satisfied these requirements and was utilized as a benchmark for assessing the resultant total tire mass.

### 2.1. Isotropic Study on Spoke Concepts

**Figure 3:**
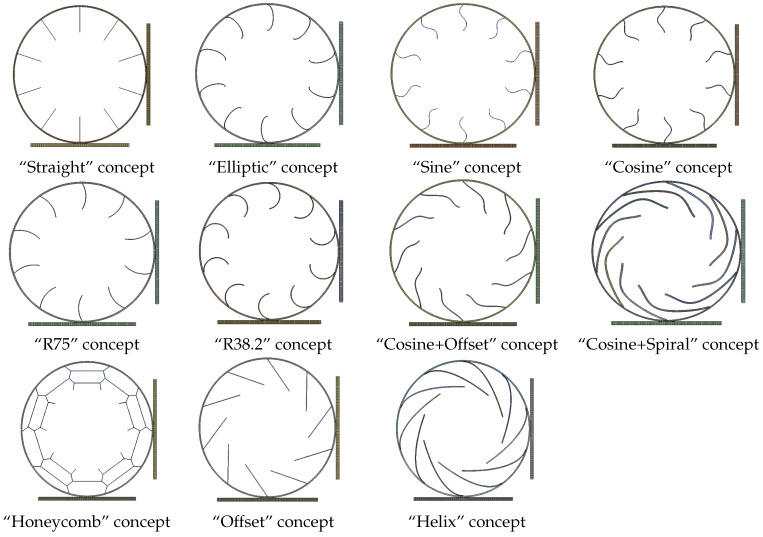
Cross-section of the analyzed NPT geometry variants.

Eleven different designs, shown in Figure 3, were assessed in the isotropic study. The proposed designs incorporated ten spokes (for the “Honeycomb” concept, this resulted in ten vertical segments) each, featuring a 4mm thick outer ring, a 250mm inner diameter, and a 400mm outer diameter. The model comprised two interchangeable ground planes, one horizontal and one vertical, facilitating the adjustment of the contact patch location between two spokes or directly at one spoke.

The empirical designs, namely “Elliptic”, “Sine”, “R75”, and “R38.2” exhibited distinctive characteristics. The “Elliptic” design comprised elliptically shaped spokes tangentially connected to both the outer ring and the rim. The “Sine” design introduced a single sine phase with an amplitude of 10 mm and zero passages at the outer ring, at the rim, and in between both. The “R75” and “R38.2” designs featured circular segments with radii of 75 mm and 38.2mm, respectively. The other concepts were related to the literature or theoretical background. The “Cosine” design was derived from the fact that the function of the buckling torque in a beam fixed on both sides is a cosine function [21], and its amplitude was set to 10 mm. Notably, the “Cosine” design shares similarities with the Unique Puncture-proof Tire System (UPTIS) created by Michelin [22]. The “Straight” design was inspired by the Tweel concept [23], while the “Honeycomb” design was inspired by the Resilient Technologies airless tire [24]. Moreover, the “Helix” and “Offset” designs were influenced by the Bridgestone airless tire concept [25]. The “Cosine + Offset” and “Cosine + Spiral” concepts, also rooted in the aforementioned Bridgestone design, additionally incorporated a superimposed cosine function, aiming to enhance the stress distribution based on the principles observed in the “Cosine” concept.

For the numerical modeling, the commercial finite element software Ansys® Academic Research Mechanical, Release 23.1 [26], was used. The geometry was modeled with Shell 281 (serendipity quadratic) elements with an average size of 3.14 mm. The nodes of the outer ring and spokes that overlapped were merged to connect the outer ring to the spokes. The tire width was set to 20 mm to reduce the computation time.

The Ansys Material Library [26] was used for the mechanical properties of the used materials. For all simulations, the spoke thickness was adjusted so that, at a vertical displacement of 20 mm, a vertical reaction force between 150 N and 175 N was achieved. For the 200 mm wide tire, this resulted in an equivalent vertical stiffness of $75\;\frac{N}{\mathrm{mm}}$ to $87.5\;\frac{N}{\mathrm{mm}}$. It is important to note that the spoke thickness was adjusted to ensure a proper comparison of the concepts based on their vertical stiffness. To reduce the influence of the varying vertical stiffness of each concept to a minimum, the result variables were later normalized by the vertical reaction force.

**Figure 4:**
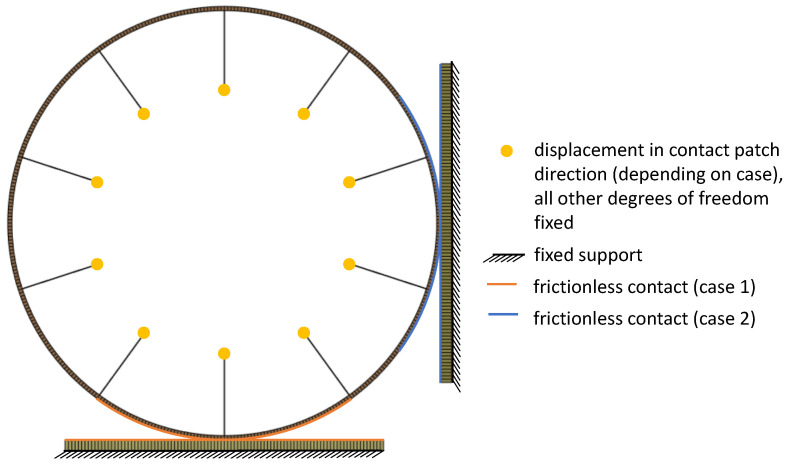
Modeling setup. Case 1 is vertical tire travel with spoke at contact patch, case 2 with contact patch exactly between two spokes.

The ground planes were fixed in all degrees of freedom. A displacement of 20 mm was applied at the inner edges of the spokes in a vertical direction to the ground. All other degrees of freedom, including nodal rotations, were fixed at the inner edges of the spokes; see Figure 4. This approach eliminated the need to incorporate a rim structure in the analysis, simplifying the problem to a rigid rim—a reasonable assumption for most automotive applications. Furthermore, frictionless contacts linked the outer ring of the tire to the ground and were enforced between the spokes and the outer ring to prevent the penetration of spokes through the outer ring of the tire. Furthermore, given the finite displacement hypothesis and the contacts, a nonlinear solver was used to solve the finite element analyses.

### 2.2. Parametric Transverse Isotropic Study

Among the eleven designs examined in the isotropic study, the “Cosine design” and the “Spiral superimposed with cosine design” were selected for a more in-depth investigation through a parametric transverse isotropic study. The rationale behind choosing these particular designs is discussed in Section 3.1. A comprehensive exploration of the parameter influences is exclusively provided for the most promising design conception in Section 3.2.

**Figure 5:**
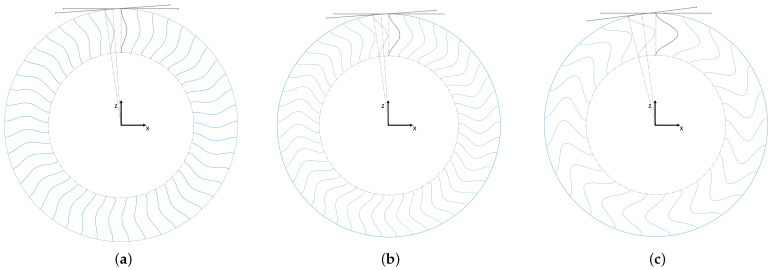
“Cosine” concepts with (**a**) a = 5 mm, (**b**) a = 10 mm, and (**c**) a = 20 mm.

For the cosine design, shown in Figure 5, three different amplitudes, *a*, were chosen for the cosine function: 5 mm, 10 mm, and 20 mm. The spokes’ designs were equation-driven using Equation (1), where *x* and *z* denote Cartesian coordinates (see Figure 5) and *t* is a curve parametrization parameter. The coordinate origin was situated in the center of the tire. The highest amplitude of the cosine function was set to 20 mm because higher amplitudes would lead to spoke collisions under load.

(1) $$\begin{array}{cc} x(t)=a\;\left[\cos\left(2\pi \;t\right)-1\right] & a=\left\{5,\;10,\;20\right\}\\ z(t)=76.2\;t+127 & t=\{t\;\in \;R\;|\;0\;\leq \;t\;\leq \;1\} \end{array}$$

where *t* is the parametrization of the curves and *x* and *z* are measured in millimeters.

**Figure 6:**
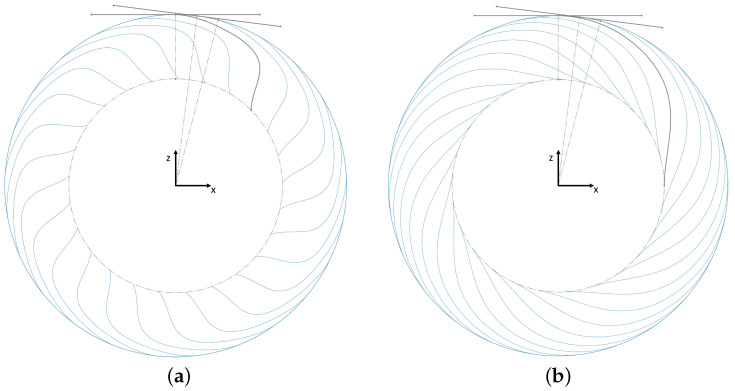
Both “Cosine + Spiral” concepts together with their spoke design equations. (**a**) “Spiral45” and (**b**) “Spiral90”.

For the spiral (superimposed with cosine) design, two spoke geometries were examined: (i) a 45-degree offset and (ii) a 90-degree offset between the inner and outer edges of the spokes with reference to a coordinate origin in the center of the tire. They are depicted in Figure 6, accompanied by their respective empirical spoke design equations (in Equation (2), the parameters are equal to those in Equation (1)). Hereafter, these configurations will be identified as “Spiral45” and “Spiral90” for ease of reference.

(2) $$\begin{array}{ccccc} x(t) & =\frac{1}{\sqrt{2}}\left(203.2-76.2\;t\right)\;t^{0.6}-10\cos\left(2\pi \;t\right)+10 & & x(t) & =\left(203.2-76.2\;t\right)\;t^{0.6}-10\cos\left(2\pi \;t\right)+10\\ z(t) & =\left(203.2-76.2\;t\right)\cos\left(\frac{\pi }{4}t\right) & & z(t) & =\left(203.2-76.2\;t\right)\cos\left(\frac{\pi }{2}t\right)\\ t & =\{t\;\in \;R\;|\;0\;\leq \;t\;\leq \;1\} & & t & =\{t\;\in \;R\;|\;0\;\leq \;t\;\leq \;1\} \end{array}$$

On the material side, CFRPs and GFRPs were used. The material data were taken from Cuntze and Freund [27] and are shown in Table 2, where φ corresponds to the fiber volume content. Furthermore, $E_{\|}$ and $E_{⊥}$ define the elastic moduli in the fiber direction and parallel to the fiber direction, respectively, while $G_{⊥\|}$ and $G_{⊥⊥}$ determine the in-plane and out-of-plane shear moduli, respectively. $\nu_{⊥\|}$ and $\nu_{⊥⊥}$ are the Poisson coefficients relative to the fiber–matrix and matrix–matrix relations, respectively. The strength of the composite is defined by $R_{\|z}$, $R_{\|d}$, $R_{⊥z}$, $R_{⊥d}$, and $R_{\|⊥}$: the fiber direction tensile, the fiber direction compression, the tensile parallel to the fiber direction, the compression parallel to the fiber direction, and the shear in the plane perpendicular to the fiber direction, respectively. The selection of FRP material data from ref. [27] ensured the good predictability of the Cuntze model, which was used for the failure criteria.

Other fibers could have been considered within the framework of this study. For instance, basalt fibers demonstrate superior performance in most Life-Cycle Assessment parameters compared to carbon and the majority of glass fibers. Additionally, flax fibers, known for their intrinsic damping properties, are among the best performing technical natural plant-based fibers [28,29]. However, given that basalt fibers exhibit mechanical responses similar to those of glass fibers and flax fibers show slightly lower performance levels [29], the selection in this parametric study was limited to carbon and glass fibers to ensure the study remained focused and methodologically coherent.

Apart from the distinctive materials—Table 2—and the specific spoke designs—Figure 5 and Figure 6—in addition to Equations (1) and (2), the parameters under examination also encompassed the following:

- The number of spokes: varied between 15 and 25;
- The thickness of the outer ring: ranged from 1.5mm to 6mm for GFRPs and 1mm to 4mm for CFRPs;
- The fiber orientation: set at 0, 15, or 30 degrees relative to the longitudinal direction;
- The thickness of the spokes: tailored for each concept to achieve the prescribed vertical tire stiffness.

The simulation model was based on the same concept as the isotropic study from Section 2.1. This meant that the parametric studies were also carried out with a 20 mm vertical displacement. Linear shell elements (Shell181 in Ansys [26]) with an element size of 5 mm were used to reduce the computing time.

For the assessment of the tire’s vertical stiffness, the force reaction arising from nodal displacement served as the output parameter. Additionally, the failure mode concept (FMC) was employed to determine the maximum inverse reserve factor (IRF) according to Cuntze and Freund [27]. The parameters utilized in the FMC are detailed in Table 3.

The 20mm displacement applied in the parametric study corresponded to 1400 N–2200 N for all design points, which fulfilled the vertical stiffness requirement of $70\;\frac{N}{\mathrm{mm}}-110\;\frac{N}{\mathrm{mm}}$. However, the strength requirements demanded a vertical load of 2956kN, which is higher by a factor of 2.1 compared to 1400N, which was achieved at $70\;\frac{N}{\mathrm{mm}}$ vertical stiffness and 20mm vertical tire travel. Hence, an upper limit of an IRF of 0.4 was deemed significant for selecting failure-proof design points for further discussion.

### 2.3. Verification Analysis

In the verification analysis, an exemplary design concept was derived, showing a possible workflow for choosing a stackup. Therefore, the following procedure was used:

1. Select, from the set of the parametric study, the lightest concept with an IRF lower than 0.4 AND the vertical stiffness requirement ($70\;\frac{N}{\mathrm{mm}}-110\;\frac{N}{\mathrm{mm}}$);
2. Perform a verification analysis using the Formula Student load cases (see Table 1);
3. Check for IRFs higher than 1;
4. If any IRF is higher than 1, exclude the concept from the set and repeat procedure 1, considering the next lightest design.

The analysis was performed with the original tire width of 200 mm. Furthermore, the mesh was populated with 10 mm sided linear elements (Shell181).

**Figure 7:**
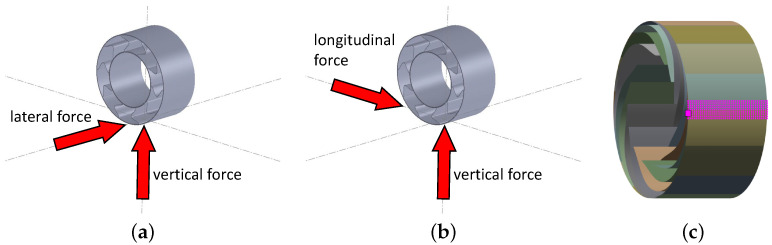
(**a**) Lateral and (**b**) longitudinal load cases and (**c**) node selection for load application (bottom view).

For the lateral load case, a lateral force of 3.71 kN and a vertical force of 2.11 kN were applied to the tire (see Figure 7a), while for the longitudinal load case, the forces were 2.60 kN and 1.95 kN in the longitudinal direction and in the vertical direction (see Figure 7b), respectively. For these load cases, the contact between the tire and the ground was not modeled, since sliding over the ground would generate a dynamic problem. To apply the loads, the contact patch was approximated with the selected set of nodes shown in Figure 7c; purple dots represent the nodes of the patch, which made up $12^{\circ }$ of the tire outer ring.

For the vertical load case, the tire was pressed against the ground until a 2.96 kN vertical force reaction was reached. The same load case was repeated for a tire with a $3^{\circ }$ camber. All load cases were examined for the tire having one spoke exactly at the bottom and for the tire having the ground exactly between two spokes. The results presented herein are only for the worst load case.

## 3. Results

### 3.1. Isotropic Study on Spoke Concepts

**Figure 8:**
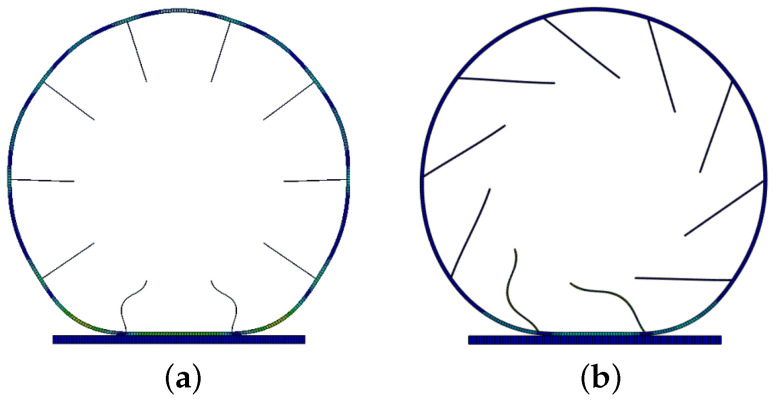
Deformed (**a**) “Straight” and (**b**) “Offset” concepts, showing different spoke buckling directions when the contact patch is between the spokes, which therefore could cause snap-through.

As result parameters, the maximum von Mises stress and the average reaction force for each concept were used. However, before comparing the concepts with each other, they were examined qualitatively. In this process, an issue with the “Straight” and “Offset” spoke concepts was observed. Both exhibited impractical buckling behavior of the spokes, illustrated in Figure 8. The buckling deformation was directed away from the contact patch on both sides, both preceding and following the contact patch. Dynamically, this behavior implies a point where the spokes would need to change their buckling direction, potentially posing challenges during high-speed driving. This snap-through problem could provide a plausible explanation for Michelin’s decision not to implement the Tweel concept with straight spokes in the Unique Puncture-proof Tire System (UPTIS).

In Table 4, the spoke thickness ($t_{s}$), the volume of ten spokes ($v_{s}$), the maximum von Mises stress ($\sigma_{max}$), and the average force reaction at 20 mm of vertical displacement ($F_{z}$) are shown for all the simulated concepts. $F_{z}$ is calculated by the mean of the force reaction with the contact patch at a spoke and with the contact patch between the spokes. In order to enhance the comparison, three different parameters were computed considering the vertical force reaction at 20 mm of vertical displacement. They were the following:

1. $\frac{F_{z}}{\sigma_{max}}$: the reaction force normalized by the maximum von Mises equivalent stress—the load-carrying capacity of the NPT;
2. $\frac{F_{z}}{\sigma_{max}\;\left(v_{s}+v_{o}\right)}$: the reaction force normalized by the maximum von Mises equivalent stress weighted by the inverse of the sum of the outer ring ($v_{o}$) and spoke volumes—the load-carrying capacity of the NPT weighted by the total mass (without the rim);
3. $\frac{F_{z}}{\sigma_{max}\cdot v_{s}}$: the reaction force normalized by the maximum von Mises equivalent stress weighted by the inverse of the spoke volume—the load-carrying capacity of the NPT weighted by the spoke mass alone.

From the results in Table 4, the following findings can be deduced:

- The spoke thickness ($v_{s}$) needs to be in a range of 0.5 mm and 4.4 mm to achieve the required stiffness range;
- The best performing concept regarding the load-carrying ability in relation to the spoke mass and total mass (second and third parameters of the list above, respectively) of the concepts examined here was the “Straight” concept due to the high amount of load being carried through the top spokes of the tire. The top spokes featured an even stress distribution, since they were mainly loaded with tension, resulting in a low maximum stress at a minimal spoke volume. However, due to buckling, the only irregularly loaded spokes were at the bottom, with their stress being low due to the thin required spoke thickness compared to that of the other concepts.
- The best performing concept regarding the absolute load-carrying ability was the “Cosine + Spiral” concept (first parameter of the list above), because it offered the lowest equivalent stress. However, it also had the highest mass of the examined concepts.
- Disregarding the “Straight” and “Offset” concepts due to their inconvenient buckling behavior, the best performing concept regarding the load-carrying ability in relation to the spoke mass was the “R75” concept, due to its low spoke mass and moderately high peak stresses.
- Disregarding the “Straight” and “Offset” concepts, the best concept regarding the load-carrying ability in relation to the total mass was the “Cosine” concept, due to its moderate peak stresses at a moderate spoke mass. It outperformed the “Offset”, “Sine”, “Elliptic”, “R38.2”, and “Honeycomb” concepts in all of the three examined parameters due to the better stress distribution in the spokes, which was caused by its shape.

As a result of the isotropic study of the spoke concepts, the “Cosine” and “Cosine + Spiral” concepts were selected for further investigation in the next chapter, since they were the best concepts in terms of the “load-carrying capacity” ($\frac{F_{z}}{\sigma_{max}}$) and “load-carrying capacity per total mass” ($\frac{F_{z}}{\sigma_{max}\;\left(v_{s}+v_{o}\right)}$), respectively. The “Cosine + Spiral” concept will be named from now on as just “Spiral”.

### 3.2. Parametric Transverse Isotropic Study

The outcomes of the cosine design study revealed that there was no IRF lower than 0.6 within the design points featuring the required vertical stiffness. Due to the large number of analyses performed, the IRF results were compressed and are presented in Appendix A. Nevertheless, it was observed that elevated amplitudes of the cosine function (20mm vs. 10mm), and consequently greater spoke thicknesses (to achieve the required equivalent vertical stiffness), resulted in a lower IRF within the spokes, as long as bottom-loading behavior was dominant. This phenomenon can be rationalized through an analogy to a bending beam: longer bending beams with an equivalent bending stiffness to shorter ones can sustain a greater load at the same stiffness, owing to their increased thickness. With small cosine amplitudes (5mm), this was not the case anymore, because these concepts mainly featured top-loading behavior, enhancing the load-carrying ability. However, to achieve the desired vertical stiffness, the 5mm cosine concept also featured extremely thin spokes (<0.2 mm), which are infeasible for manufacturing. In summary, this indicates that the load-carrying capacity of the cosine design is insufficient for non-elastomeric materials such as GFRPs and CFRPs.

Similar results for the IRF (>0.4) were observed with the configuration “Spiral45”. The values of the IRF, along with the parameters tested, can also be found in Appendix A. On the other hand, the “Spiral90” design showed inverse reserve factors as low as 0.3. Consequently, a detailed analysis of the parameter influences was undertaken for this design only.

#### 3.2.1. Spoke Thickness

**Figure 9:**
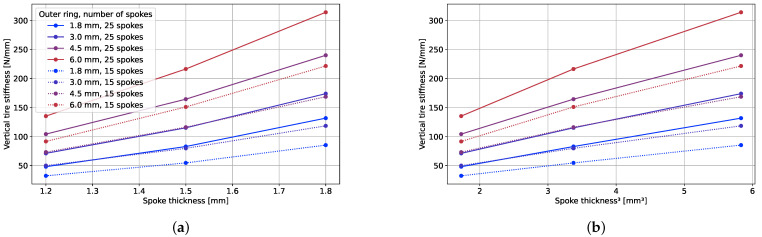
GFRP stackups’ vertical stiffness × (**a**) spoke thickness and (**b**) third order of spoke thickness (proportional to moment of inertia).

**Figure 10:**
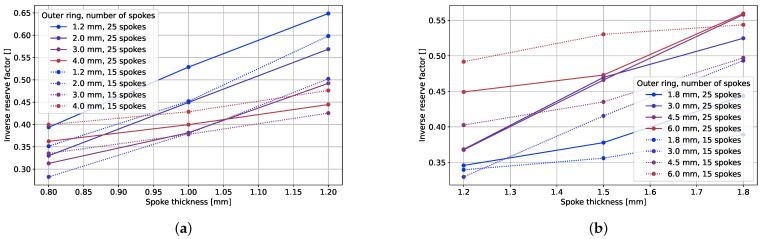
IRF × spoke thickness of (**a**) CFRPs and (**b**) GFRPs.

Figure 9 illustrates the impact of the spoke thickness on the vertical tire stiffness. An increase in the spoke thickness corresponds to an augmentation in the vertical stiffness. If the spokes are regarded as bending beams, the stiffness can be assumed to increase with the third power of the thickness. To identify a potential correlation with the NPT vertical stiffness behavior, the vertical stiffness was graphed against the third power of the spoke thickness, shown in the right plot of Figure 9. Notably, the NPT stiffness exhibited a robust correlation with the third power of thickness for thin outer rings, as indicated by the constant slope. However, for thicker outer rings, this correlation diminished, resulting in a less pronounced increase in the vertical stiffness with an increasing spoke thickness. The inverse reserve factor also increased with the spoke thicknesses, as shown in Figure 10. This can also be explained by the analogy to a bending beam: higher thicknesses lead to higher strains at an equal applied displacement.

#### 3.2.2. Outer Ring Thickness

**Figure 11:**
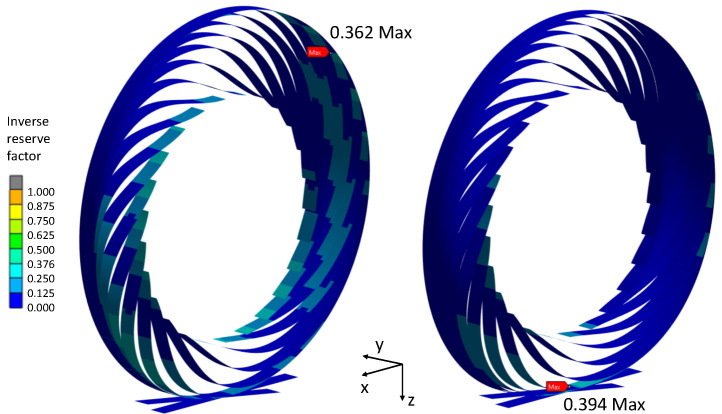
Inverse reserve factors of CFRP concepts with 0.8mm spoke thickness and 1.2mm outer ring (**right**) and 4mm outer ring (**left**).

**Figure 12:**
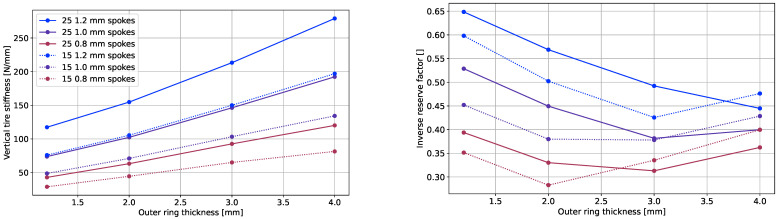
Vertical stiffness of CFRP stackups over outer ring thicknesses (**left**), and inverse reserve factor over outer ring thicknesses (**right**).

The variation in the NPT stiffness in terms of the outer ring thickness is depicted in Figure 9. Conversely, the outer ring thickness did not exert a similar influence on the IRF, as illustrated in Figure 10. In certain instances, the inverse reserve factor even diminished despite the augmentation in the vertical stiffness and consequently heightened loads on the tire. This phenomenon can be attributed to the alterations in the contact conditions between the spokes and the outer ring. Additionally, a propensity for greater top-loading behavior was observed with an increasing outer ring thickness, as evidenced in Figure 11. The substantial impact of the outer ring thickness on the vertical tire stiffness, coupled with its relatively lower impact on the reserve factor at an equal displacement, underscores its influential role in the load-carrying capacity. This observation is further substantiated by the plots in Figure 12.

#### 3.2.3. Fiber Material and Orientation

The plots in Figure 10 highlight that the GFRP concepts exhibited a lower inverse reserve factor compared to the CFRP concepts. Nonetheless, owing to the lower stiffness of glass fibers, achieving similar vertical stiffness behavior necessitated higher spoke and outer ring thicknesses. This, coupled with the higher density of glass fibers, culminated in an approximate doubling of the weight of glass fiber concepts compared to their carbon fiber counterparts.

Another parameter for GFRP or CFRP NPT design is the fiber orientation. It was found that fiber directions other than 0^∘^ have adverse impacts on the tire vertical stiffness as well as the IRF. This information is also found in Appendix A.

#### 3.2.4. Number of Spokes

The plots in Figure 12 reveal that higher spoke numbers contributed to an increased load-carrying ability if concepts with a similar vertical tire stiffness were compared. This is also shown in Table 5, which compares the mass and inverse reserve factor for carbon fiber concepts with equal outer ring thicknesses and a comparable vertical stiffness across various spoke numbers and thicknesses. Nevertheless, it was observable in all cases that for a similar vertical stiffness, few thicker spokes resulted in lighter NPTs than thinner ones in a larger quantity. This might suggest a possibility of optimizing the number of spokes, along with their thickness, to achieve a lighter version of an NPT.

### 3.3. Verification Analysis

**Figure 13:**
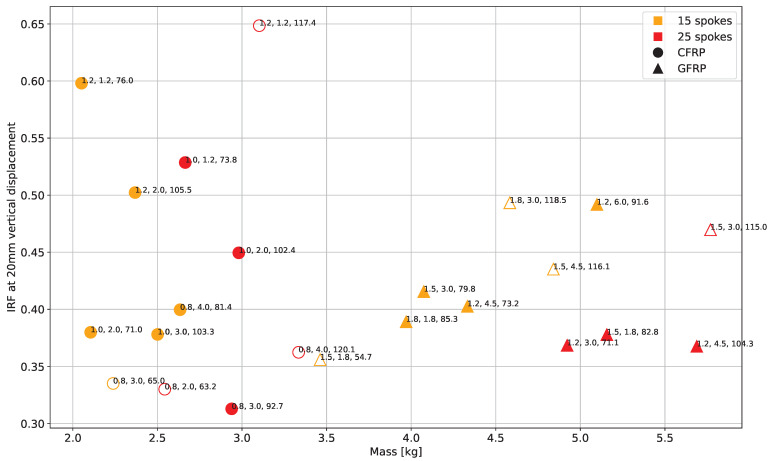
NPT IRF × mass at 20mm vertical displacement with $50\;\frac{N}{\mathrm{mm}}$ to $130\;\frac{N}{\mathrm{mm}}$. Each point shows spoke thickness [mm], outer ring thickness [mm], and vertical stiffness [N/mm], respectively, where full markers correspond to designs that were within vertical stiffness requirement (see Table 1) and outlined-only markers to those outside.

The plot in Figure 13 illustrates all concepts with vertical stiffnesses ranging from $50\;\frac{N}{\mathrm{mm}}$ to $130\;\frac{N}{\mathrm{mm}}$ and zero-degree plies. Care should be taken when quantifying the results from this graph, since a different vertical stiffness resulted in various loads at 20mm vertical tire travel, and therefore the IRFs are not easily comparable. Still, it underlines the findings from Section 3.2, namely the connection of the fiber material, number of spokes, and outer ring thickness to the tire mass and IRF.

For choosing a final concept, the procedure presented in Section 2.3 was used. This yielded the 15-spoke CFRP design with a 3 mm outer ring and 1 mm spoke thickness, being the lightest feasible concept at 2.5 kg. However, keeping manufacturing difficulties, e.g., achieving the desired fiber volume content, or changes in geometry, e.g., corner radii, in mind, this is to be considered only as a suggestion. Still, due to the mass reduction of 1.1 kg compared to the *Hoosier FSAE 16.0X7.5-10* pneumatic tire, which comes in at 3.6 kg, and also the fact that state-of-the-art NPTs are much heavier than their pneumatic counterparts, this shows that there is potential to lower the tire weight with the incorporation of FRPs. The results of the verification analysis are summarized in Table 6.

## 4. Discussion

The conducted studies revealed that the use of straight spokes in NPTs would be the preferred option, yet their buckling behavior in different directions from the contact patch renders them unsuitable for high-speed applications. This is supported by the fact that NPTs with this kind of tire are only available for slow-moving vehicles. Excluding the concept with straight spokes, the concept with the most favorable load-carrying behavior in relation to the total tire mass was found to be the one featuring cosine-shaped spokes. This design reduces the bending moment in the bottom spokes, thereby enhancing the load-carrying capability through the top spokes. A key takeaway from this is that for NPT design, the aim should be to find a spoke concept with low bending stiffness, so that the bending stress is low, and high tensional stiffness, so that the top spokes can carry the load through tension, which loads the spokes much more uniformly and also involves more spokes in the load-carrying process compared to bottom-loading NPT concepts.

However, in the studies involving FRPs, it was found that even the cosine-shaped spokes exhibit excessive strain for use with CFRPs and GFRPs. Consequently, the design with spiral spokes, superimposed by a cosine, was chosen for further detailed examination. Despite being a bottom-loading concept, the spiral design demonstrated the highest load-carrying ability among the examined concepts. Nevertheless, it also exhibited the highest mass, contradicting the original intention of achieving weight savings through the utilization of CFRPs or GFRPs for NPTs. However, due to the extreme lightweight potential of FRPs, a total mass reduction of 30% compared to the reference pneumatic tire could still be achieved, emphasizing the potential of fiber-reinforced tire structures.

For the spiral design, it was found, among other things, that thicker outer rings increase the stiffness with only a marginal impact on the reserve factor at the same tire travel. Therefore, thick outer rings should be introduced to enhance the stiffness with flexible spokes, ultimately augmenting the load-carrying capacity of NPTs. However, according to the literature, the contact pressure is expected to increase with stiff outer rings, especially if they are not designed as shear beams [14]. Since the contact pressure was not examined in this study, no further statement can be made about this subject. It should be noted, however, that fiber-reinforced designs offer the flexibility to use different matrices [30], potentially providing low shear stiffness for the design of an outer ring shear beam.

Further findings on the proposed spiral design were that the spoke can be seen as a bending beam since an increase in the thickness increases the stiffness by approximately the third power. This, however, likely applies primarily to bottom-loading tires, as top-loading spokes are not subject to bending but rather tension. Given that FRPs, especially CFRPs and GFRPs, are too stiff concerning the bottom spokes but simultaneously suitable for creating high tensile stiffness in the top spokes, a fiber-reinforced elastomer tire emerges as a potential solution, warranting future exploration to reduce the weight of NPTs. Nonetheless, it was demonstrated that a non-elastomeric NPT made from CFRPs can meet critical tire requirements, exhibiting low vertical stiffness while maintaining a sufficient load-carrying ability.

## 5. Conclusions

This study presents the development of NPT concepts based on continuous fiber-reinforced epoxy composites as an alternative to traditional elastomer-based designs. A structured methodology was implemented, involving finite element modeling to evaluate different spoke configurations, followed by a parametric study considering the material anisotropy and load-bearing capabilities. The study aimed to enhance the tire performance while reducing the overall weight, ensuring structural integrity under multiple load conditions.

- The geometric evaluation identified cosine-shaped spokes as featuring the highest load-carrying ability in relation to the tire mass and a spiral design superimposed by cosine spokes as the most efficient in minimizing the material stress, when considering the multiple concepts tested.
- The material selection demonstrated that CFRPs offer significant weight reduction compared to pneumatic tires while maintaining structural durability.
- The load-bearing behavior analysis indicated that the spiral design with a 90-degree offset provided optimal stress distribution and sufficient resilience under various load conditions.
- The outer ring thickness played a crucial role in improving the design safety; however, dynamic tests and the contact pressure still need to be analyzed in future work, most likely limiting the outer ring thickness.
- The manufacturing feasibility highlighted the need for the further exploration of fiber-reinforced elastomeric composites to mitigate potential brittleness and improve flexibility.

While being a preliminary parametric study, this research confirms the potential of continuous fiber reinforcement in NPTs. Still, as aforementioned, further studies should investigate the dynamic behavior of composite materials under real-world driving conditions, including the rolling resistance, impact absorption (e.g., through a drum–cleat test), contact pressure, and long-term durability. Future work will also explore hybrid materials—in terms of different fibers and matrices—that combine the lightweight benefits of composites, paving the way for next-generation NPT technologies that prioritize both performance and sustainability.

## Figures and Tables

**Table 1:** Chosen tire specifications based on Formula Student evaluation [20].

Type of Requirement	Parameter	Physical Value
	Rim diameter	247 mm–250 mm
Geometric	Outer tire diameter	400 mm–406.4 mm
	Tire width	200 mm–203.2 mm
Stiffness	Vertical stiffness	70 N/mm–110 N/mm
	Longitudinal load case	2.60 kN longitudinal, 1.95 kN vertical
Strength	Vertical load case 1	2.956 kN vertical
	Vertical load case 2	2.956 kN vertical with 3 degrees of camber
	Lateral load case	3.71 kN lateral, 2.11 kN vertical

**Table 2:** Material data of the used GFRPs and CFRPs [27].

Material Type	φ [%]	$E_{\|}$ [GPa]	$E_{⊥}$ [GPa]	$G_{⊥\|}$ [GPa]	$\nu_{⊥\|}$ [-]	$\nu_{⊥⊥}$ [-]	$G_{⊥⊥}$ [GPa]	$R_{\|z}$ [MPa]	$R_{\|d}$ [MPa]	$R_{⊥z}$ [MPa]	$R_{⊥d}$ [MPa]	$R_{\|⊥}$ [MPa]
GFRP	60	45.60	16.20	5.83	0.278	0.400	5.79	1280	−800	40	−145	60
CFRP	60	138.00	11.00	5.50	0.280	0.400	3.93	1500	−900	27	−200	80

**Table 3:** FMC parameters for calculation of IRF according to Cuntze and Freund [27].

Parameter	Value
Rounding-off exponent, *m*	2.6
In-plane shear friction, $\mu_{⊥\|}$	0.2

**Table 4:** Overview for comparison of examined NPT spoke concepts.

Concept		$t_{s}$ [mm]	$v_{s}$ [cm^3^]	$\sigma_{\max}$ [MPa]	$F_{z}$ [N]	$\frac{F_{z}}{\sigma_{\max}}$	$\frac{F_{z}}{\sigma_{\max}\left(v_{s}+v_{o}\right)}$	$\frac{F_{z}}{\sigma_{\max}\;v_{s}}$
Straight	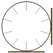	0.5	7.5	116	155	1.33	0.0123	0.178
Offset	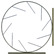	1.6	36.5	174	172	0.99	0.0072	0.027
Elliptic	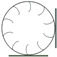	1.5	26.5	207	164	0.79	0.0062	0.030
Sine	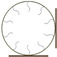	1.4	24.3	190	170	0.90	0.0072	0.037
R75	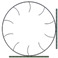	1.2	18.8	181	152	0.84	0.0070	0.045
R38.2	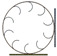	2.2	46.3	153	174	1.13	0.0077	0.024
Cosine	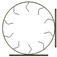	1.8	31.2	148	172	1.17	0.0089	0.037
Honeycomb	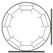	0.8	49.0	218	167	0.77	0.0051	0.016
Helix	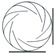	3.0	129.4	95	164	1.72	0.0075	0.013
Cosine + Offset	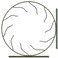	2.1	50.5	126	158	1.26	0.0084	0.025
Cosine + Spiral	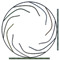	4.4	203.8	68	169	2.48	0.0081	0.012

The last three columns have dimensions; however, these hold no specific significance.

**Table 5:** Comparison of 15- and 25-spoke CFRP tires with a similar vertical stiffness in each row. The tires with 25 spokes featured a lower inverse reserve factor but also a higher mass compared to the ones with 15 spokes.

Outer Ring Thickness [mm]	25-Spoke Concept				15-Spoke Concept			
	Spoke Thickness [mm]	Vertical Stiffness [N/mm]	Inverse Reserve Factor	Mass of 200 mm Wide Tire [kg]	Spoke Thickness [mm]	Vertical Stiffness [N/mm]	Inverse Reserve Factor	Mass of 200 mm Wide Tire [kg]
1.2	0.8	42.8	0.39	2.2	1.0	48.7	0.45	1.8
1.2	1.0	73.8	0.53	2.7	1.2	76.0	0.60	2.1
2.0	0.8	63.2	0.33	2.5	1.0	71.0	0.38	2.1
2.0	1.0	102.4	0.45	3.0	1.2	105.5	0.50	2.4
3.0	0.8	92.7	0.31	2.9	1.0	103.3	0.38	2.5
3.0	1.0	146.2	0.38	3.4	1.2	150.0	0.43	2.8
4.0	0.8	120.1	0.36	3.3	1.0	134.2	0.43	2.9
4.0	1.0	192.3	0.40	3.8	1.2	197.0	0.48	3.2

**Table 6:** Summary of load cases and their IRFs.

Load Case	Lateral	Positive Longitudinal	Negative Longitudinal	Vertical	Vertical with Camber
Vertical load [N]	2.109	1.946	1.946	3.206	3.036
Lateral load [N]	3.713	0	0	0	0
Longitudinal load [N]	0	2.604	−2.604	0	0
Peak IRF	0.93	0.79	0.88	0.55	0.74

## Data Availability

The raw data and numerical models supporting the conclusions of this article will be made available by the authors on request.

## Appendix A

This appendix shows the IRF of several different NPTs. These values were used for the decision-making process that defined the “Spiral90” as the chosen concept for further studies. For the sake of brevity, in the tables in the appendix, $t_{o}$ and $t_{s}$ define the outer ring and spoke thicknesses in mm, respectively, while α corresponds TO the fiber angle in degrees. The vertical stiffness is determined by $V_{s}$ in N/mm. Furthermore, the tables present these values for both CFRPS and GFRPS. For some concepts, more configurations are shown for one material than the other. This is due to the fact that not all configurations achieved the vertical stiffness range defined by the stiffness test in Table 1.

**Table A1:** Concept Cosine20.

				25 Spokes		15 Spokes						25 Spokes		15 Spokes	
	$t_{o}$	$t_{s}$	α	$V_{s}$	IRF	$V_{s}$	IRF		$t_{o}$	$t_{s}$	α	$V_{s}$	IRF	$V_{s}$	IRF
CFRPs	3	0.4	0	83.6	0.71	57.7	0.71	GFRPs	4	0.6	0	85.2	0.94	59.3	0.85
	4	0.4	0	104.8	0.70	70.0	0.71		2	0.8	0	84.8	1.23	58.2	1.10
	4	0.48	15	115.7	1.63	79.4	1.48		3	0.8	0	120.0	1.27	83.7	1.14
	4	0.6	30	100.8	2.58	70.1	2.43		4	0.8	0	155.4	1.28	108.7	1.15
	2	0.48	0	85.1	0.88	59.5	0.82		1	1.0	0	109.7	1.49	65.2	1.27
	3	0.48	0	122.7	0.86	85.7	0.84		2	1.0	0	143.3	1.57	93.8	1.35
	4	0.48	0	157.5	0.84	107.7	0.85		1	1.2	0	173.7	1.70	104.0	1.52
	1	0.6	0	85.06	1.15	53.4	0.88		4	0.8	15	133.7	1.93	93.4	1.82
	2	0.6	0	136.1	1.12	94.8	1.00		4	1.0	30	152.0	3.39	105.8	3.07
	1	0.72	0	137.0	1.38	83.5	1.05								

**Table A2:** Concept Cosine5: manufacturing is not feasible due to the extremely thin spokes.

				25 Spokes		15 Spokes						25 Spokes		15 Spokes	
	$t_{o}$	$t_{s}$	α	$V_{s}$	IRF	$V_{s}$	IRF		$t_{o}$	$t_{s}$	α	$V_{s}$	IRF	$V_{s}$	IRF
CFRPs	1	0.08	0	15.8	0.40	14.0	0.40	GFRPs	2	0.12	0	39.8	0.77	35.8	0.75
	2	0.08	0	116.7	0.63	105.7	0.60		3	0.12	0	129.0	0.97	117.4	0.94
	1	0.12	0	19.2	0.55	16.0	0.55		2	0.16	0	41.9	0.90	37.0	0.88
	2	0.12	0	119.5	0.71	107.7	0.70		3	0.16	0	131.2	1.10	118.9	1.07
	1	0.16	0	25.6	0.72	20.3	0.72		2	0.2	0	45.6	1.03	39.1	1.00
	2	0.16	0	125.6	0.85	111.2	0.84		3	0.2	0	134.6	1.22	121.0	1.19

**Table A3:** Concept Cosine10.

				25 Spokes		15 Spokes						25 Spokes		15 Spokes	
	$t_{o}$	$t_{s}$	α	$V_{s}$	IRF	$V_{s}$	IRF		$t_{o}$	$t_{s}$	α	$V_{s}$	IRF	$V_{s}$	IRF
CFRPs	2	0.3	0	74.8	0.95	54.2	0.89	GFRPs	4	0.4	0	94.5	1.18	70.2	1.12
	3	0.3	0	118.9	0.95	88.4	0.90		3	0.5	0	103.6	1.46	74.7	1.39
	1	0.4	0	75.3	1.26	48.6	1.19		4	0.5	0	142.0	1.48	103.8	1.41
	2	0.4	0	130.5	1.26	93.6	1.19		1	0.6	0	74.0	1.54	44.3	1.45
	1	0.5	0	134.6	1.59	82.8	1.49		2	0.6	0	101.3	1.65	70.0	1.60
									3	0.6	0	147.8	1.75	105.9	1.66
									4	0.5	15	121.8	2.21	88.8	2.10
									4	0.6	30	122.4	3.46	88.3	3.32

**Table A4:** Concept Spiral45.

				25 Spokes		15 Spokes						25 Spokes		15 Spokes	
	$t_{o}$	$t_{s}$	α	$V_{s}$	IRF	$V_{s}$	IRF		$t_{o}$	$t_{s}$	α	$V_{s}$	IRF	$V_{s}$	IRF
CFRPs	4	0.4	0	71.601	0.519	47.348	0.557	GFRPs	6	0.6	0	81.179	0.625	53.673	0.666
	2	0.6	0	81.8	0.49	59.7	0.47		3	0.8	0	73.7	0.58	54.0	0.53
	3	0.6	0	126.6	0.54	92.4	0.59		4.5	0.8	0	114.5	0.60	83.4	0.67
	1	0.8	0	87.9	0.63	60.7	0.63		6	0.8	0	152.5	0.71	107.2	0.77
	2	0.8	0	145.6	0.66	106.5	0.66		3	1	0	114.3	0.72	83.2	0.66
	4	0.6	15	122.1	1.10	87.6	1.22		6	0.8	15	130.6	1.09	91.7	1.21
	4	0.8	30	110.9	1.57	82.1	1.75		6	1	30	142.7	1.64	103.4	1.83
